# Hypodermic Use of Iodine in Goitre

**Published:** 1870-04

**Authors:** 


HYPODERMIC USE OF IODINE IN GOITRE.—Prof. Lücke (*Braithewaite's Retrospect*) treats goitre by injecting hypodermically four or five drops of tincture of iodine, and repeating the injection every eight days or so, gradually increasing the quantity.—*St. Louis Med. Archives.*

